# Slipping through the net: a qualitative study exploring women’s experiences of maternity in the UK during the COVID-19 pandemic

**DOI:** 10.1186/s12884-026-08913-9

**Published:** 2026-03-10

**Authors:** Freya J Harding, Anne Gatuguta, Alice Chi Eziefula

**Affiliations:** 1 Royal Sussex County Hospital, Eastern Road, Brighton and Hove, Brighton, BN2 5BE UK; 2 Department of Global Health and Infection, Brighton and Sussex Medical School, 94 N – S Road, Falmer, Brighton, BN1 9PX UK

**Keywords:** COVID-19, Pregnancy, Maternity, Social support, Qualitative, Pandemic

## Abstract

**Background:**

The quality of maternity experiences impacts the health and wellbeing of both the mother and baby. Widespread changes were made to maternity care and social support during the COVID-19 pandemic in the UK, altering the usual experience of pregnancy, birth and postnatal care. Remote care appointments and hospital/home visiting restrictions affected the extent to which women could receive medical, social, and emotional support. The aim of this study was to explore women’s experiences of pregnancy, birth, and the postnatal period during the COVID-19 pandemic in South East England.

**Methods:**

In-depth interviews were conducted with 12 participants who had given birth during the COVID-19 pandemic. Participants were recruited from mother and baby groups and data were analysed thematically.

**Results:**

Women felt isolated and under-supported during the perinatal period. This was most noted in the postnatal wards by women who experienced immobilisation and pain following caesarean section. Reported positive experiences were improved opportunity to bond with their child and avoidance of undue external child-rearing pressures and pregnancy-related negative stories. Telemedicine was considered useful for some participants, and felt unsafe for others. Innovative use of social media to share up-to-date information improved crucial communication.

**Conclusions:**

Changes in staffing and visiting restrictions contributed to unsatisfactory experiences at home and on postnatal wards. The study highlights shortcomings in pandemic maternity services, and the importance of planning for future critical incidents, to promote positive maternal and child health outcomes. Telemedicine and social media have the potential to improve care and foster a sense of community in maternity services, however, further evaluation to ensure safety is needed.

**Supplementary Information:**

The online version contains supplementary material available at 10.1186/s12884-026-08913-9.

## Background

The Coronavirus disease 2019 (COVID-19) pandemic forced many changes to the way women experience pregnancy, childbirth, and the postnatal period in the United Kingdom (UK) and globally. The World Health Organization (WHO) declared a pandemic in March 2020 and the UK activated a national lockdown shortly afterwards. The UK remained under some form of social restriction until July 2021 [1]. Significant changes were implemented in the operations of the National Health Service (NHS), including maternity services [2], to reduce the risk of COVID-19 transmission; to redirect resources to COVID-19 patients; and to respond to the expanding number of patients who were critically ill [3]. These changes included the use of telemedicine (where care is delivered remotely) for midwifery and doctors’ appointments. Visiting restrictions were implemented during appointments, birth and in postnatal wards, and COVID-19 polymerase chain reaction (PCR) test status affected triage and bed allocation.

Beginning in March 2020, women faced differing levels of restrictions on their social movements, which influenced their ability to socialise with friends and family, to meet other pregnant women, and to form new friendships [4]. Pregnant women were predicted to be more vulnerable to severe COVID-19 [5], evidenced subsequently in the increase in maternal deaths attributable to COVID-19 during 2020–2022 [6]. As a result, on 16th March 2020, before a national lockdown began, the UK government included pregnancy as a defining characteristic of clinical vulnerability to COVID-19 [5], with the consequent advice to follow social distancing measures more stringently and to work from home where possible, particularly for those in high-risk jobs, such as healthcare.

Changes to hospital care, delivered by the NHS, were swift and varied across different hospitals and regions of the UK [2, 7]. There was a reduction in face-to-face appointments, appointments were to be attended alone, and the number of birth partners and the duration of time partners could spend with women in the hospital were reduced [2]. Options for birthing places were reduced: home births, water births, alongside midwifery units (attached to a hospital obstetrics ward but midwife led) and free-standing midwifery units (not at a large hospital site) were all unavailable at various points throughout the pandemic [2]. Staffing changed significantly. A survey of junior doctors working in Obstetrics and Gynaecology showed that 53% were redeployed to other services [8].

Quality of experiences of prenatal care, birth and postpartum care is important. It can influence physical and mental health outcomes of women and children and affects child development [9–13]. Positive birth experiences are more likely when women feel emotionally supported, in births with minimal intervention, and in women who are well-prepared for birth [14–16]. Women who have continual emotional support during childbirth are less likely to require interventions during birth, and babies are more likely to be born with a higher Appearance, Pulse, Grimace, Activity, Respirations (APGAR) score [17]. Birth preparation is sourced through NHS services, independent antenatal groups, such as the National Childbirth Trust and increasingly popular hypnobirthing services, and through informal learning networks of friends and family, all of which were disrupted or moved online [14]. Experienced control, a state that can be achieved when women select their intended birth environment, by choosing where they want to give birth, deciding who they want to be present at birth, and choosing their approach to pain relief, is also a significant predictor of birth satisfaction [18]. High levels of control are associated with high levels of birth satisfaction [18]. The mandatory adjustments made during the pandemic facilitated a feeling of a loss of control for many women.

Negative perinatal experiences increase the risk of chronic maternal and infant morbidity. Predictors of antenatal mental health conditions include lack of social support, adverse life events and heightened levels of stress [19]. In comparison to pre-pandemic period, a global review documented higher prevalence levels of anxiety (30.5%) and depression (25.6%) in pregnancy during the COVID-19 pandemic; with evidence of increasing prevalence of anxiety as the pandemic progressed [20, 21]. This highlights an increase in potential risk of poor maternal and child outcomes as both pregnancy anxiety and depression increase the risk of obstetric complications such as preterm birth and the risk of postnatal depression [10].

Moreover, very poor or traumatic experiences at birth increase the risk of mental health conditions, such as anxiety, postnatal depression (PND), and post-traumatic stress disorder (PTSD) [22–24]. Childbirth-related PTSD can have long-lasting effects on women’s health and on relationships with their children and partners [13]. Positive birth experiences are a strong predictor of a positive postnatal experience [11, 22]. In the context of the significant changes in maternity services, our study aimed to document women’s experiences of pregnancy, birth, and the postnatal period during the COVID-19 pandemic in the UK. This research contributes to the understanding of the impact on women and maternity services of the COVID-19 pandemic and adds to qualitative analyses of maternity care during COVID-19 [25].

## Methods

Women who gave birth during the pandemic were purposively recruited from antenatal groups and postnatal mother and baby groups in South East England. The study aimed to recruit a diverse population including women from different ethnicities and socioeconomic backgrounds. The groups were contacted to participate and a recruitment poster which included ethnically diverse women representation was sent. The groups included free to join groups as well as groups where members paid a subscription fee. The inclusion criteria were open to all ethnicities, ages (> 18), socioeconomic status and education.

Twenty-eight groups were identified, and the group organisers were approached as gatekeepers. Twenty-two groups agreed to participate and study information was shared through a study poster. Potential participants were invited to contact the lead researcher, who screened participants for eligibility. The eligibility criteria were: (1) Is pregnant or has given birth since 11/03/2020 until time of recruitment (30/06/2021) (2) Aged over 18, (3) Resident in the UK from pregnancy onwards, (4) No current diagnosis of a mental health condition or debilitating medical condition.

If the eligibility criteria were met, a participant information sheet was emailed to them with a consent form. The participant information sheet explained who the researchers were, what the purpose of the study was, any potential benefits and disadvantages of taking part, and how confidentiality would be maintained at all times including; storing of their data securely with only the researchers having access to it; their data recording and transcripts being anonymised; and the option to withdraw from the study at any point. After reading the participant information sheet, the participants gave their informed consent by completing the consent form and emailing it back to the lead researcher.

Data were collected through in-depth, semi-structured interviews. In line with social distancing guidelines at the time, interviews were conducted on Zoom video conferencing software and lasted between 90 and 120 minutes. An interview guide was used to explore topics such as social support, access to care, and communication about changes to care. The interview guide was created for this study and has been provided as supplementary file 1. Interviews were carried out until the point at which no new ideas or relevant information was being obtained during interviews was reached.

The interviews were audio-recorded and transcribed verbatim. The information was anonymised in the transcription process; each participant was allocated a number: P1-P12 and personal identifiers were removed from the data. Thematic analysis was employed by the research team using both inductive and deductive approaches [26]. The research team consisted of three researchers; the lead researcher was a medical student at the time of the interviews and is now a doctor with an interest in obstetrics and gynaecology; the other two are experienced researchers with expertise in women’s health, infectious diseases, (including research and clinical expertise in COVID-19), and qualitative research methods [27–34].

Rigour was upheld in the analysis by having a clear procedure of creating codes and themes. Deductive codes were developed in line with the overall aim of the study (pregnancy, birth and postnatal experiences) while new codes were identified inductively through recurring patterns within the data. The codes and code book were created by the lead researcher and then reviewed and discussed as a group. Through this process, code names were refined until the final codes were agreed upon. Next, the codes were grouped into initial themes by the lead researcher followed by a consensus discussion. Disagreements about the codes and themes between the researchers were discussed and resolved by reviewing the original data to align the codes and themes as closely as possible to the primary data. NVIVO 12 software was used to organise the data during thematic analysis. Accurate and detailed records and a reflexive journal were kept by the lead researcher throughout the data gathering process.

Ethical approval was granted by Brighton and Sussex Medical School Research Governance and Ethics Committee: ER/BSMS9A83/1 on 25/03/2021. An amendment to the application to collect more demographic information: ER/BSMS9A83/1/1 was approved on 28/04/2021.

### Reflexivity statement

The lead researcher (interviewer) was a medical student at the time of interviews, and this may have influenced how they approached the research and interpreted the data. The researcher was cognizant of this and therefore maintained a reflexive approach throughout. Being a medical professional put the researcher in a trusted position; however, the researcher made the participants aware that they were a student and carrying out research which would have no immediate impact on the participant’s care. They were also able to recognise, understand and empathise with the seriousness of the medical complications participants and their babies experienced, which participants appreciated, and this may have led to more disclosures (both positive and negative).

Furthermore, there may be an over representation of participants’ medical issues and encounters with medical professionals, as this was the most familiar ground to the researcher, so further questioning and detail on these topics was easily explored. The participants may have wanted to share more medical information as they knew the researcher may be particularly interested in these topics. To ensure a balanced approach, the researcher used a pre-prepared interview guide developed collaboratively with the other two researchers which included pre-conceived probes alongside exploring emerging issues. The researcher also emphasised they wanted to explore both clinical and non-clinical experiences. Throughout the study, the researchers held reflective discussions acknowledging their positionalities and how this may influence the research.

## Results

Twelve women from South East England were interviewed. All the women gave birth between May 2020 and Feb 2021; a time period that spanned a range of social restrictions from “full lockdown” to targeted social distancing. Three participants had given birth previously, adding comparative aspects to their experiences. All participants who agreed to participate were in heterosexual relationships, identified as women, lived with their partners and were white British, except one British national of Asian heritage. This relative homogeneity of the participants may have contributed to data saturation being reached with a modest sample size.

All women had A-Level education (a post-secondary school qualification for university or career progression in the UK), and eight had degree-level education. Participants’ occupations included nursing, medicine, radiography, law, teaching, and language translation. All participants gave birth in a hospital, except one participant who had a home birth but was transferred to a hospital shortly after birth for neonatal care support.

Overall, most participants had challenging experiences related to the quality of care and support provided by professional healthcare providers, family and social contacts such as friends, antenatal and baby groups. Although the majority of the experiences were negative, women also reported some unexpected benefits from the social restrictions imposed. The findings are presented below under six identified themes. Theme 1: Impact of changes in social structure, Theme 2: Impact of changes to healthcare services, Theme 3: Quality of communication from healthcare professionals, Theme 4: Self-reported traumatic experiences, Theme 5: Challenges of childcare with reduced support, Theme 6: Unexpected benefits of parenting during the pandemic.

### Impact of changes in social structure

Family and friends, especially mothers, may provide emotional support and practical help during the perinatal period. The limitations on social contact due to pandemic restrictions meant that many women felt lonely and isolated. Participants often said that they had an idea that this time should be a joyous and happy one, but instead, it was sad and lonely. They expressed sadness at missing a shared experience with their families and friends during their pregnancies, with their newborn babies, and as their babies grew and developed.

*P2: If we’d have been able to join an NCT [National Childbirth Trust] group*,* we’d have other mums and dads we could message*,* but we didn’t have that support because it was online.*

*P1: I do think they [family] missed out so much in my pregnancy.*

Pregnant women were advised to stay at home and ‘shield’ by the government, before the rest of the country was put into lockdown. This put women in an anxious situation; being among the first people to experience change in their social and working environment.

*P11: I felt anxious when [the then UK prime minister] said vulnerable people should stay at home [shield]*,* and if you’re pregnant*,* you’re vulnerable to COVID-19.*

Working women faced drastic changes to their work lives because of the risk of severe COVID-19 in pregnancy. Many participants were essential workers and five worked in the NHS. Some participants were advised to change roles and/or work from home given their vulnerable status, due to concerns that they might become infected with COVID-19 at work. However, they also felt conflicted as this led to feelings of isolation and loneliness.

*P3: …was somebody going to get COVID and then give it to me? I was dreading going to school. I think if I’d done that [worked from home] the whole time*,* I would have been quite lonely…I was a bit torn in that.*

*P8: It was quite isolating being at home*,* there’ll be days where I wouldn’t speak to anybody for a couple of days.*

### Impact of changes to healthcare services

Most antenatal groups were cancelled towards the beginning of the pandemic. Instead, some women attended online classes and found some benefits, such as not having to travel. Most women expressed that they would have preferred to attend in-person groups and that they missed out on forming meaningful friendships as a result.

*P6: That was something I was looking forward to* [*referring to baby groups*], *but then…it all closed down…it’s more about meeting the other mums and dads… he* [the baby] *has no little friends*,* which is a bit sad.*

Participants who had given birth previously felt the impact of a lack of baby groups more keenly. They highlighted not being able to get out of the house, to meet other mums, and learn about baby care. One participant links the development of postnatal depression to the lack of groups.

*P4: I went to lots of groups with my previous baby; stay and plays*,* paid ones*,* free ones. They were for different ages*,* under 6 months*,* under 12 months*,* over 12 months. Professionals would come in to talk about sleep training*,* weaning*,* teething. I didn’t get to do anything like that with him…I got postnatal depression*. *I think*,* had groups been running*,* I would have been alright: I’m getting out of the house; I’m not gonna do the housework; I’m gonna go to a group of other mums and see them face to face. There wasn’t anything running.*

Antenatal appointments used telemedicine where possible, and companions or visitors were excluded from any necessary face-to-face appointments, including ultrasound scans. Participants reported this as a cause of significant worry for them. Women who had had complications in previous pregnancies, such as miscarriages and sepsis, felt appointments triggered more anxiety and fear in them. Not having their partners with them meant there was no one to support and comfort them through these difficult feelings.

*P6: …I’d had quite a few scares before [in previous pregnancies] I was quite nervous*,* about going for that 20-week scan…I was quite nervous*,* having had all the miscarriages.*

*P4: Because of my past experience of having sepsis following the birth of my first. Having a baby brings on all that anxiety. To not have him there for that reassurance*,* was difficult. I kept panicking*,* is everything okay?*

For support, some participants connected with their husbands or partners via speaker phone during in-person appointments. They expressed a preference for face-to-face visits for all appointments. Some participants observed that their partners seemed detached from their pregnancy as a result of these restrictions. Most participants opted to pay for private scans, as their partners were allowed to attend these. However, this caused uncertainty as to what underlaid the different recommendations in the public and private sectors.

One participant gave birth when most social restrictions had been removed. She valued the fact that she was able to attend all her appointments in person with her partner, highlighting the importance of a supportive companion.

*P11: We were just lucky. If my partner couldn’t come to the 12-week scan*,* I would have been a mess. And the same for the 20-week scan…if there is something wrong*,* that’s when you need someone there.*

### Quality of communication from healthcare professionals

At the beginning of the pandemic, hospital correspondence and communication were perceived as unclear. Women reported a sense that hospital staff seemed to be unsure of the recommendations and perceived this to be due to the evolving nature of the pandemic.

*P2: The communication was hard*,* and I think part of the difficulty wasn’t their fault*,* so people just didn’t know what was going to be happening.*

*P12: [I asked] Do you need to wear a mask all the time [in hospital]? Some said yes*,* and some said no.*

Clear communication about changes to the care structure was important to women. Some anxieties about childbirth stemmed from not knowing whether, or for what duration, their partners would be able to attend the birth. A lack of clear communication led to difficulty in following instructions. For example, one participant reported that she did not self-isolate before her planned caesarean section as this requirement had not been communicated to her and she only became aware of it upon arrival at the hospital.

Communication improved as the pandemic progressed. Participants who had babies later in the pandemic reported good communication from their hospital about COVID-19 guidelines, mostly in letter format. Most women were able to confirm instructions directly with their midwives by contacting them on a mobile phone number. Social media, such as Instagram^®^, was used by some midwifery units to provide instant updates about guideline changes, which women found useful, and accessible, designating it as the preferred mode of communication.

*P9: I think most of it was announced*,* so if you follow social media pages*,* it’s kinda where they kept you up to date.*

Two women who had an elective (planned) caesarean section observed that, knowing when and how they were going to give birth and knowing that their partner could be present, reduced their levels of anxiety.

### Self-reported traumatic experiences

Some participants described their experiences of childbirth and postnatal care as emotionally traumatic. Trauma during childbirth was related to a lack of personal support and sub-standard communication. The rule that partners could only be present when women were in active labour (cervical dilation >5 cm), meant that some participants spent long periods labouring alone in understaffed maternity wards; an experience they found especially difficult. The use of personal protective equipment (PPE) made the environment hostile to participants and led to challenges in communication between staff and participants. Participants reported that the stressful environment and restrictions on labour wards made the experience of birth complications even more traumatic for them. A participant who had an emergency caesarean section highlighted this thus:

*P1: …the one that upset me the most was the COVID… But it was all those restrictions on top of it that*,* that’s what made it difficult*,* not how he arrived [her newborn son*,* by emergency caesarean section] It was everything else on top that made it difficult.*

*P3: When people came in to see me*,* they were wearing their kind of full PPE…looked like they didn’t want to be in with me.*

Negative and traumatic experiences were also related to a lack of contact and communication due to inadequate staffing levels, to the restrictions on visiting partners and family to nurture and care for women, and to prolonged separation from newborn babies immediately after birth because of infection prevention control measures. One participant reported having to wait six hours for a negative COVID-19 test before she could meet her newborn baby. Postnatal ward visiting rules ranged from strictly no visitors to visiting for up to four hours per day. This allocation was inadequate for women, who wished to celebrate the birth of their child with partners and relatives and receive essential connection and support from them. Overall, many participants felt uncared for, lonely, isolated and unable to look after their babies on the postnatal ward.

This was particularly challenging for women following caesarean section. Post-operatively, women had to deal with the effects of spinal anaesthesia and abdominal incision. Both made picking up their babies extremely difficult. In ideal situations, they would have had a partner with them to help or they would have used a call buzzer to request a staff member to assist. Under the circumstances of reduced staffing and staff redeployment, waiting times to get assistance were longer. Some women reported having waited for long periods for staff to attend, causing them to resort to less-than-ideal ways of reaching their babies.

*P6: I was in a lot of pain for a long time afterwards [after the caesarean section]*,* getting him [the baby] out of his cot*,* I had to drag him by the feet.*

Two participants reported being diagnosed with post-traumatic stress disorder (PTSD) after these experiences.

*P2: I experienced PTSD after that [the birth and stay on the postnatal ward]*,* which manifested in flashbacks regularly.*

The significance of this trauma was underscored by the fact that participants filed formal complaints and expressed a strong desire to ensure that something like that did not happen again.

*P10: I also did put a complaint in against the aftercare… so I hope that it does make some sort of difference if this were to ever happen again…*.

### Challenges of childcare with reduced support

Poor breastfeeding support was cited as a major issue. On the postnatal ward, a participant reported being shouted at by a midwife to “get on with” breastfeeding, instead of being helped. This participant put in a complaint about the experience, and in response, they were advised that, usually, there are breastfeeding support workers on the wards to assist when mothers have breastfeeding problems, but these were stopped during the pandemic as they were not seen as essential, and therefore posed a risk of COVID-19 exposure, and that the midwife may have shouted due to time pressures and increased workload on the wards.

Challenges in postnatal care after discharge from the hospital were most notably related to breastfeeding issues. Standard care in the UK involves home visits by midwives on days 1, 5 and 10 after discharge. Some participants reported that these appointments were conducted either using telemedicine, were cancelled, or sometimes missed without explanation. Usually, midwives monitor babies’ weights, mother’s breastfeeding techniques and output, and signs of dehydration during the scheduled visits. One participant, who was struggling to establish breastfeeding, believed that telemedicine appointments may have contributed to her baby being readmitted to the hospital with dehydration. The baby was crying inconsolably, yet when she discussed this on the telephone with the midwife, she was told, ‘Babies just cry.’ Eventually, a midwife paid a home visit and identified instantly that the baby was severely dehydrated and needed hospital admission.

Without these face-to-face appointments, participants felt that a responsibility was forced upon them, to identify adverse clinical signs in their babies. They had to do this without the tools and expertise available to healthcare professionals, such as baby weighing. Participants felt this was unsafe.

*P11: I couldn’t tell she’d lost 15% of her body weight. I couldn’t tell that by looking at her. And I do think … that physical*,* somebody in my home is so important. I felt like I slipped through the net.*

Breastfeeding hotlines, a telephone number that women could call if they were having breastfeeding problems, were available throughout the pandemic. In general, most participants found them unhelpful because of the sheer physicality of breastfeeding; being given instructions over the phone was not sufficient and could even be dangerous. The participant in the example above used a breastfeeding hotline. The remote consultation provided false reassurance to her.

Telephone appointments placed women in a difficult situation when they were asked to assess the clinical condition of their babies. Women felt that this approach was inappropriate because they did not have the skills or understanding to assert confidently that their babies were healthy, nor the courage to voice their concerns.

*P3: You’re trying to describe that over the phone [a new rash on her baby]…I don’t have a medical background.*

*P4: Doesn’t make it personal enough for you to say “Actually no*,* I’m a bit worried about that”.*

Baby weighing clinics were disrupted, which interfered with growth monitoring. Some women reported that health visitors would ask the mothers if they had any concerns about the baby’s weight, but mothers found it difficult to know if they had concerns without first weighing the baby.

*P4: There wasn’t any weighing clinics…Well*,* I don’t know what my concerns are*,* until he gets weighed… we’ve gone and got ourselves a baby scale.*

### Unexpected benefits of parenting during the pandemic

The pandemic caused social norms to be disrupted. Participants found that the social distancing restrictions allowed them a level of control as to who visited them soon after childbirth.

Some women reported relief at a reduced volume of counsel and information they received from family, friends, and strangers, relating to pregnancy and birth. One woman appreciated a pregnancy and postnatal experience with the absence of shared difficult birth stories and unsolicited advice about pregnancy and caring for a newborn baby.

*P11: Nobody was giving me their horror stories of their horrible labours. And I had a really large amount of control over the information that I had… I didn’t have unsolicited advice from strangers.*

Furlough schemes and working from home allowed partners to spend more time with women when they were pregnant and when the baby arrived. Participants reported that partners were able to form a strong bond with the baby.

As in-person social events were cancelled, some women were more accepting of the isolation of new motherhood. They enjoyed the ability to focus on their baby without any expectation or invitation to socialise before they were ready.

The maternity triage service, an emergency phone line service for pregnant women, was perceived to be effective and supportive of women during the pandemic. They felt it was easy to access; providers responded promptly and with appropriate concern and escalated to an in-person review when necessary. There was limited alteration to these services during the pandemic.

Some participants saw value in access to telemedicine for straightforward routine appointments. They felt this provided a medically appropriate review that was supportive of their needs. Telephone appointments saved time and money on travel and were easier to schedule into work and personal lives.

*P12: If everything’s going to be routine and straightforward*,* it’s nice if there is an option for telephone or video. I think it works well.*

Similarly, the use of technology to socialise during the pandemic was viewed positively.

*P2: It’s meant that we’ve seen each other a lot more than we ever used to [through video calls]…thank goodness we’re doing this in an age where technology exists.*

## Discussion

Participants in this study had difficult experiences of maternity during the COVID-19 pandemic. Many felt isolated, unsupported, and alone at times when they most needed support from friends, families, partners, and healthcare professionals. This resulted in stress and emotional trauma, factors known to contribute to negative health outcomes for both mother and baby [35–39]. Our study findings show that a reduced sense of control and support impacted the quality of birth experiences. This aligns with other published studies that documented negative experiences during the COVID-19 pandemic [25, 40, 41]. Our study highlights some less-documented findings and lessons, including positive experiences early in the baby’s life due to increased time dedicated to uninterrupted maternal-child bonding; the perceived impact of telemedicine/remote maternal and child services; and the importance of family support in the context of limited staffing levels in maternity services.

Current evidence suggests that the pandemic maternity service environment may have increased the risk of women having traumatic birth experiences during the pandemic. While our study cannot draw causal links between the pandemic and the incidence of reported postnatal PTSD, the findings align with evidence from an Italian study (a country that undertook similar levels of restriction) that found 42.9% of postpartum women in the study reported symptoms of PTSD [42], compared to a pre-pandemic rate of 3–4% [24]. Our findings highlight other risk factors that may contribute to PTSD and other mental health problems such as anxiety, uncertainty, loss of control, subjective negative birth experiences, and lack of social support [20, 42, 43]. These findings underscore the importance of putting in place mechanisms to address these factors when implementing extensive changes in care provision during national emergencies.

Participants who had planned caesarean section, perceived that they had more positive birth experiences^,^ due to a sense of control over the situation, in keeping with established findings that women who have higher perceived levels of control during birth report their experiences more positively [44]. Perceived levels of control, the ‘extent to which a mother believes her actions influenced or shaped the conditions of the birth environment’ are highly predictive of the quality of birth experiences [45]. Greater perceived control during birthing creates more positive emotions, decreases fear and guilt, and increases satisfaction in care, especially in first-time mothers [44]. Ways in which control in birth is experienced, such as choosing where to give birth, were removed as options. This may have presented a higher likelihood of dissatisfaction with maternity care, which was reported frequently in our study.

Evidence suggests that women who have social support during childbirth have shorter labour, better pain control, and less need for medical intervention [17]. This is recognised in WHO and National Institute for Health and Care Excellence (NICE) guidelines for birthing mothers [46, 47]. Continuous support throughout labour is found to have the highest impact on improving birth outcomes [17] and decreases the incidence of PND [48]. All the participants in this study had their partners present only for the active stage of labour. These restrictions meant there was a significant period during which women were labouring on their own. In the UK, the incidence of PND increased from 16% in 2018 to 23.9% in 2020 [49].

Social support during pregnancy and the post-natal period facilitates women’s adaptation to motherhood and is recognised in international and national policy as necessary for maternal and infant well-being. A lack of peer support in pregnancy and postnatal periods due to decreased access to antenatal and baby groups is reported to make these experiences more isolating and difficult for women [41]. Often under-recognised, this study highlights the value of personal social support in the postnatal ward settings. Our study highlights the feeling of helplessness that women felt without their partners, family members and friends present to take care of them and their babies in the face of limited staffing. Collectively, these experiences demonstrated that postnatal wards rely to some extent upon care from partners and family members and that, without them, women receive inadequate support.

Similar to findings in other studies, women perceived that, in the pandemic setting, the level of care that ward staff could provide was inadequate [50]. Pre-pandemic it was estimated there was already a national shortage of 2500 full-time midwives in England [51]. This situation was worsened during the pandemic due to redeployment, absences due to COVID-19 infection and isolation of contacts, thus approaching a risk of falling short of a minimum level of staffing to deliver sufficient care [52].

While not new, the application of telemedicine in the NHS, grew exponentially during the COVID-19 pandemic and its use has continued subsequently. Instigated due to the prioritisation of infection control, a need for cost-effectiveness by avoiding unnecessary hospital visits, and leveraging the widespread technology available, this mode of care is likely to continue. Services offered traditionally by doctors in face-to-face consultations, such as prescriptions and referrals are now more commonly offered remotely. Evidence suggests that telemedicine has similar outcomes to in-person care, in some settings [53, 54]. However, there are safety concerns in maternity care. One maternal death has been linked to its use during the pandemic [55]. Providers of maternal healthcare have expressed concerns over the impact on quality of care and the lack of guidelines related to telemedicine [56]. A realist review of digital consultations in maternity has detailed implementation principles for establishing effective use of telemedicine, the ‘CORE’ principles, but notes data on safety and equity is still lacking [57]. The current evidence, and our study findings, suggest that some elements of maternity services may be offered safely and effectively through telemedicine, but more research is needed.

The WHO recommends “Effective communication between maternity care providers and women in labour, using simple and culturally acceptable methods” to achieve person-centred outcomes [46]. The experiences of the women in this study suggest communication was impaired during the pandemic period, and this affected their overall experience of birthing. Social media use was cited as supportive of communication between maternity service providers and participants, suggesting a demand for a responsive, health information and advice service on non-urgent issues for pregnant women. Other authors have found that social media can be used to create a sense of ‘virtual community’ and provide social support [58, 59]. These findings warrant further research on how the use of social media can be adopted in routine care and future emergency situations.

### Limitations

Although the recruitment strategy aimed to enhance diversity and inclusion, this study explored the experiences of a sample of women with similar socioeconomic backgrounds. In order to recruit women from diverse backgrounds the study team contacted perinatal care group gatekeepers across the whole of South East England, including groups across a range of access costs, including free ones. The study materials were designed for accessibility and for relevance to women from diverse racial and cultural backgrounds. On reflection, the design of the study poster and contacting a broad range of groups was not sufficient to recurit a diverse sample of women. Addressing additional barriers to participating in research such as access to groups; time and resources to participate in groups and research; as well as targeted recruitment of under-represented groups and Patient and Public Involvement stakeholder consultations, may improve inclusion in future research.

The interviewed participants were relatively homogenous in demographics such as education level, jobs, ethnicities, ages, and partnered status which likely influences the diversity of their experiences of maternity in the pandemic. Evidence suggests that higher education levels, being white, being partnered and a high socioeconomic status are associated with better experiences of maternity care [60, 61]. Thus this study may have under-represented adverse or traumatic birth experiences during the pandemic. Having necessary resources, links and experience of working in health services may have enabled this group to access alternative care and services such as online antenatal classes more easily. Despite this, the findings demonstrate that this was not enough to protect them from the negative impact of the changes the COVID-19 pandemic imposed on their medical care and social lives. Our study did not capture the experiences of less educated, single, younger and women from ethnic minorities whom evidence shows are at a higher risk of poorer maternity care and outcomes [60–63].

Women with difficult or traumatic experiences may have been overrepresented in this study due to the nature of voluntary recruitment. A common reason for participation in the research was that the women found a part of their experience traumatic, and they wanted to participate to contribute feedback to the health service, so that experiences could be improved for women in the future. This is a reported phenomenon in birth experience research and may contribute to experiences of women with satisfactory or routine experiences not being represented in research [62]. Many studies of birth experience investigate, record and quantify negative experiences in birthing. A small proportion of studies investigate positive birth experiences. The estimated prevalence of negative birth experiences globally is 16% of births [64], suggesting that negative birth experiences could be over represented in research themes.

Our study included five participants who worked within the NHS in various roles. Although not evident, their experiences and perceptions of care may have been influenced by their professional knowledge and roles within the healthcare system.

## Conclusion

Women felt inadequately supported both emotionally and physically and experienced a lack of control over their birthing experience during the pandemic. Routine maternity care processes that ensure safe monitoring and response mechanisms for the mother and baby were changed, leading to a risk to women of slipping through the net. Reduced connection with partners and professionals whilst accessing care during pregnancy, birth and the postpartum period at the hospital precipitated traumatic experiences. However, pandemic restrictions brought unexpected positive effects, such as, an increase in the uninterrupted time mothers and partners spent with their babies, and the preservation of the quality of access to emergency care. Prioritising positive pregnancy, birth and postnatal experiences is necessary, even during national emergencies, to promote the health and wellbeing of the mother and baby, which have long-term implications.

Evaluation of experiences during the COVID-19 pandemic may hold wide-reaching lessons for the development of current maternity service models as well as for future critical incident and pandemic planning. Our study highlights (1) the importance of telemedicine during a crisis and the need to investigate its safety in maternity care; (2) the need to investigate the role and safe use of social media in providing information to pregnant women, especially in emergencies; (3) the need to evaluate the role of partners and social support in perinatal care in emergency settings.

## Supplementary Information

Supplementary Material 1.

## Data Availability

The datasets used and analysed during the current study are available from the corresponding author on reasonable request.
