# Supplementary material for: Slipping through the net: a qualitative study exploring women’s experiences of maternity in the UK during the COVID-19 pandemic
Source: BMC Pregnancy Childbirth. 2026 Mar 10;26:422. doi: 10.1186/s12884-026-08913-9 (PMC13088518; doi:10.1186/s12884-026-08913-9)
Supplement: Supplementary file 1 — Supplementary Material 1. [file 12884_2026_8913_MOESM1_ESM.docx]

Interview Guide

Introduction:

Thank you for agreeing to take part in this study. My name is Freya Harding I am studying to be a doctor and have taken a year out of my medical degree to study a master’s degree in Global Health. I have some questions I would like to ask you, the questions I ask may vary depending on your answers and I may ask questions to follow up from the answers you give me. Before I begin the questioning, I would like to confirm that you understand what the study is for and that you consent to taking part. I would also like to reiterate that you can stop this interview at any time, without giving a reason. You may also ask to not answer a question or a particular topic without giving a reason. The question’s in the interview are not meant to upset you, so if they do please tell me and the interview will be stopped.

Could you tell me generally about your pregnancy, birth and postnatal time?

Theme – Social Support

Can you describe the social support you have? This can include family, friends, antenatal groups, baby groups.

Can you explain how your social support has been affected by COVID-19?

- Can you tell me about how you used online methods to stay connected with your social support networks?

What are your thoughts on pregnant women being in the shielding category at the beginning of the pandemic?

Can you tell me about how this pregnancy has been different from your previous pregnancies?

Can you tell me about your experiences of antenatal classes?

- Can you tell me about your experience of online antenatal classes?

Can you tell me about the changes to your work as a result of COVID-19 and explain what you felt about this in terms of your pregnancy?

- Can you tell me about specific changes made to your work due to your pregnancy?

**Women who have given birth:**

Can you tell me about how COVID-19 changed the support you were able to have at your birth?

Can you tell me about any mother and baby classes you attended?

- Do you think COVID-19 influenced your decision in attending the classes?
- If you attended in person, how did you feel about that?

Can you explain what the support from your family and friends been like since you gave birth?

Can you tell me about your breastfeeding experience? How do you think this is different because of covid?

Can you tell me about your bonding with you baby?

Can you tell me about your partners bonding with the baby?

Can you tell me about your baby’s social development? How has this been different because of covid?

Theme – Access to care

Can you tell me about your antenatal appointments and what your thoughts are about them?

Can you explain what your relationship with your midwife/obstetric team is like?

- How do you feel this has been affected by COVID-19?

Can you tell me about how you felt about your access to your midwife and hospital during your pregnancy?

Can you explain how you (did) feel about going into hospital?

- Can you describe your emotions about going into hospital?

Can you explain how COVID-19 affected your birthing options?

How did these changes make you feel about your birth?

**Women who have given birth:**

Can you tell me about your birth?

Can you explain your opinion on the care you received during your birth?

What was your experience of the health visitor and midwife appointments?

- Were these appointments online / telephone call?

How did these appointments make you feel?

Have you had your vaccine? - was this easy to access?

Theme – Communication regarding changes to care

How were changes to your antenatal care communicated to you?

What is your opinion of how these changes to your care were communicated to you?

Could you explain how this could have been improved?

Can you describe what your communication with your midwife was like?

How did you feel about the communication with the NHS staff during your pregnancy?

**Women who have given birth:**

Can you explain how you were told about the COVID-19 safety measures that would be in place for your birth?

- Including the use of personal protective equipment by staff and checks you would be subjected to

Can you tell me about what you thought of the COVID-19 safety measures?

- How did these make you feel?

Can you explain how the midwife and health visitor appointments were communicated to you?

Tell me about your opinion of how these were communicated to you?

Can you explain whether the appointment format was the same as how it was explained to you?

Demographic Information:

- Age:
- Where they live (County, city/town/village):
- Location of birth:
- Pregnant or delivered:
- Date of delivery:
- Number of pregnancies:
- Ethnicity:
- Religion:
- Relationship status:
- Gender:
- Sexuality:
- Profession (occupational status):
- Highest Educational attainment:
- Method of conception (spontaneous vs artificial methods):

End of Questions

Have you got anything else you would like to add?

Have you got any questions?

Thank you.
